# Supplementary material for: Topochemical synthesis of different polymorphs of polymers as a paradigm for tuning properties of polymers
Source: Nat Commun. 2020 Feb 13;11:865. doi: 10.1038/s41467-020-14733-y (PMC7018732; doi:10.1038/s41467-020-14733-y)
Supplement: Supplementary file 1 — Supplementary Information [file 41467_2020_14733_MOESM1_ESM.pdf]

Supplementary Information

Topochemical synthesis of different polymorphs of a polymer as a paradigm for tuning properties of polymers

Mohanrao et al.

Supplementary methods

1. Methods

All the chemicals were purchased from commercial suppliers and used without purification. ^{1}H spectra were recorded in a 500 MHz spectrometer. Proton chemical shifts (δ) are relative to tetramethylsilane (TMS, δ = 0.0) as internal standard and denoted in parts per million (ppm). IR spectra were recorded in an IR Prestige-21 (Shimadzu) spectrometer. Melting points were determined using a Stanford Research systems (EZ-Melt) melting point apparatus. X-ray intensity data measurements of freshly grown crystals were done on a Bruker-KAPPA APEX II CCD diffractometer with graphite-monochromatized (MoK = 0.71073Å) radiation. Refinement was carried out with SHELXL-2014. The PXRD experiments were conducted using slow and continuous scan rate mode using Cu as the anode material (Kα1 = 1.540598Å). MALDI-TOF mass spectra of samples were recorded using 2,5-dihydroxybenzoic acid as the matrix using Bruker UltrafleXtreme MALDI-TOF mass spectrometer. Gel Permeation Chromatography (GPC) experiments were carried out in DMF on an Agilent Technologies 1260 infinity GPC/SEC (PLgel column). DSC analyses were carried out using DSC Q20 differential scanning calorimeter, at a heating rate of 5 ^oC per min. TGA analyses were done using universal V 4.7A TA instrument at a heating rate of 10 ^oC per min. The crystal structures were overlaid using Mercury (version 3.10.1) software.

2. Topochemical reactions of **DP-II** and **DP-III**

Crystals of **DP-II** and **DP-III** were taken in test-tubes and placed in a pre-heated oil bath at 60 ^oC and continued heating at that temperature. The progress of the topochemical reaction was monitored by withdrawing small fractions at different times and then recording their ^{1}H NMR (DMSO-d_6), FT-IR, PXRD and DSC. Both the polymorphs underwent topochemical reaction to give the corresponding pseudoproteins **PP-II** and **PP-III**.

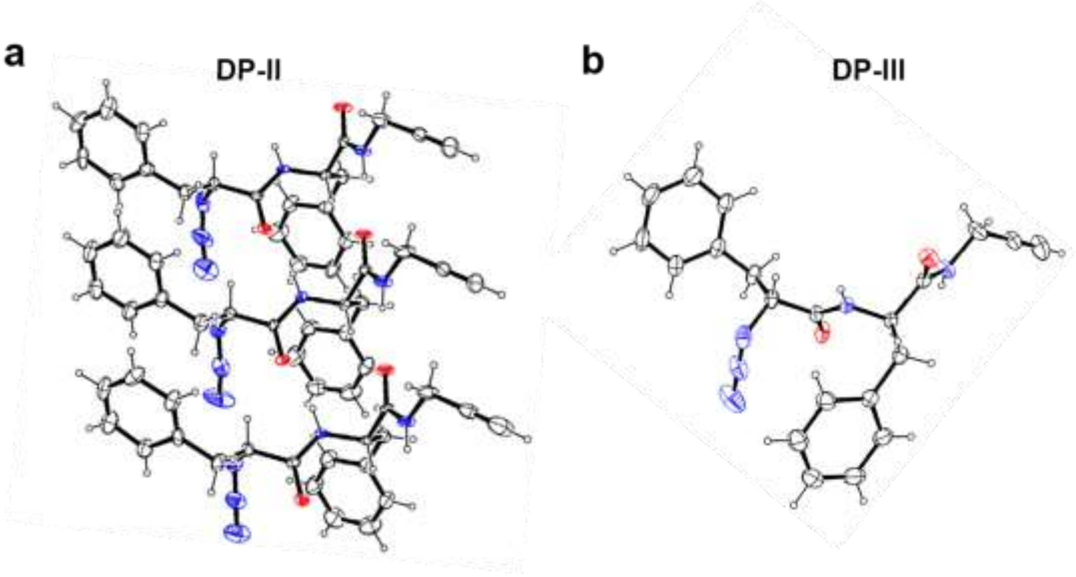

Supplementary Figure 1. ORTEP diagrams of DP-II and DP-III. **a, b** ORTEP diagrams of **DP-II** and **DP-III** respectively with ellipsoids drawn at 20% probability level.

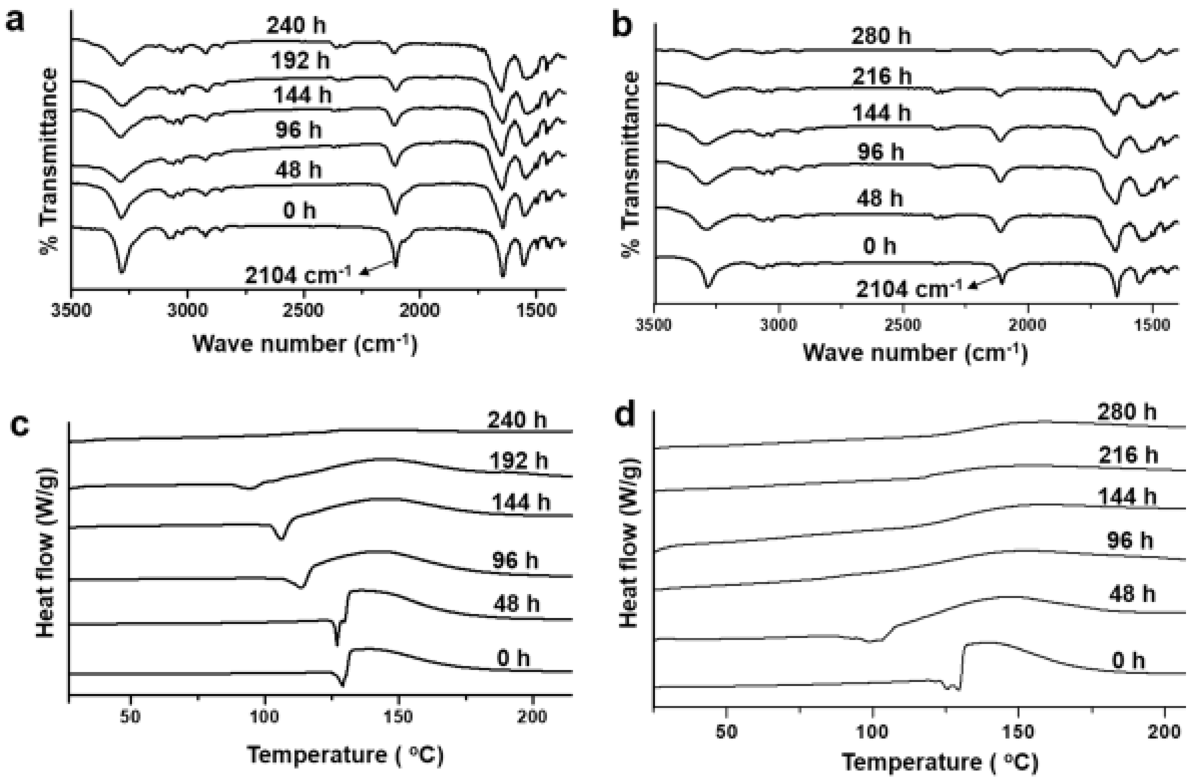

Supplementary Figure 2. **Time-dependent studies for TAAC reactions of DP-II and DP-III. a** Time-dependent FT-IR of TAAC reaction of **DP-II. b** Time-dependent FT-IR of TAAC reaction of **DP-III**. **c** Time-dependent DSC of TAAC reaction of **DP-II. d** Time-dependent DSC of TAAC reaction of **DP-III**. Source data are provided as a Source Data file for Supplementary Figs. 2a, 2b, 2c and 2d.

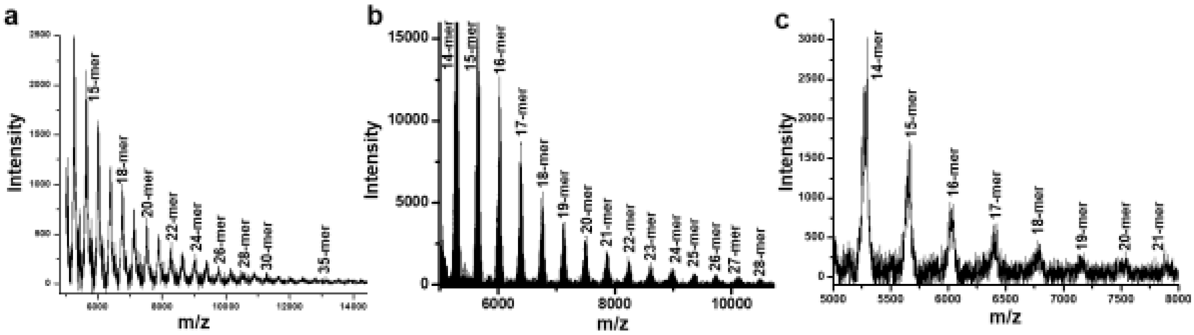

Supplementary Figure 3. **MALDI-TOF mass spectra of the pseudoproteins.** MALDI-TOF mass spectra of **PP-I** (**a**), **PP-II** (**b**) and **PP-III** (**c**). We have recorded the MALDI spectrum of the reacted samples by solid/solid sampling method.[1] The solid sample of the pseudoproteins was mixed with 2,5-dihydroxybenzoic acid (DHB matrix) in 1:1 ratio and ground together. The obtained mixtures were analyzed by MALDI-TOF spectrometry. Source data are provided as a Source Data file for Supplementary Figs. 3a, 3b and 3c.

a

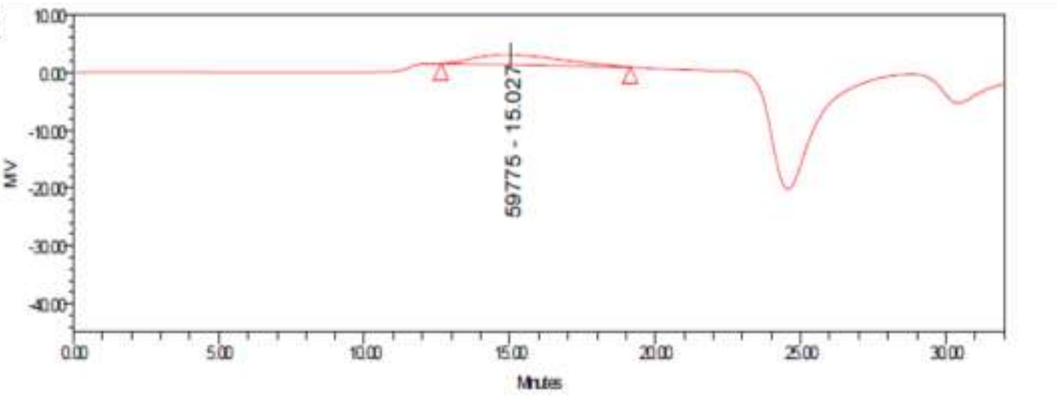

GPC Results

	SampleName	Mn	Mw	MP	Mz	Mz+1	Mv	Polydispersity
1	RS-PPI	40736	56313	59775	72508	86817		1.382384

b

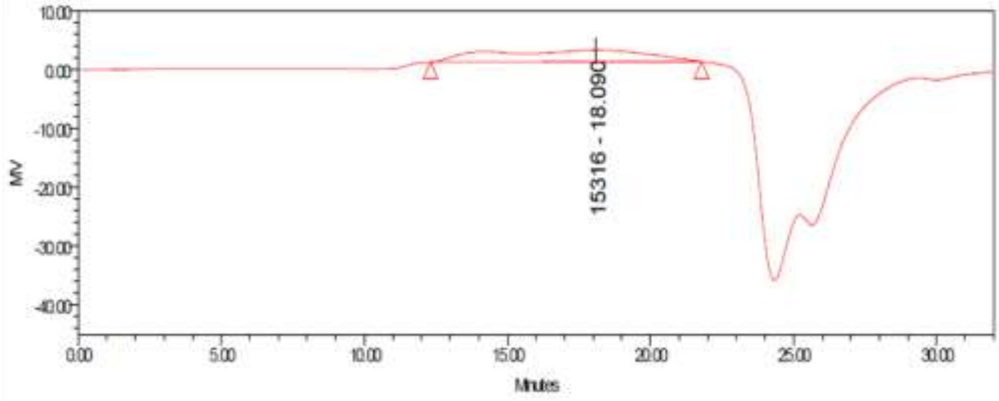

GPC Results

	SampleName	Mn	Mw	MP	Mz	Mz+1	Mv	Polydispersity
1	RS-RAJAN-PPII	16411	41118	15316	77533	102689		2.505478

c

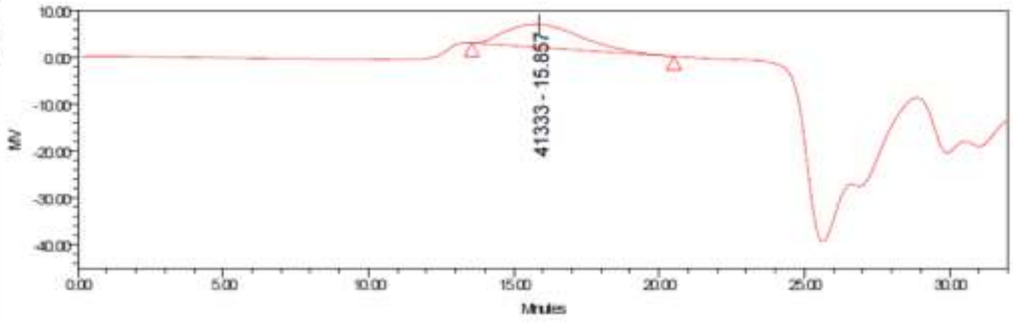

GPC Results

	SampleName	Mn	Mw	MP	Mz	Mz+1	Mv	Polydispersity
1	RS-RAJAN-PPIII	30010	39519	41333	48780	56031		1.316863

Supplementary Figure 4. Gel permeation chromatography analysis of the pseudoproteins. Gel permeation chromatograms of **PP-I** (**a**), **PP-II** (**b**) and **PP-III** (**c**).

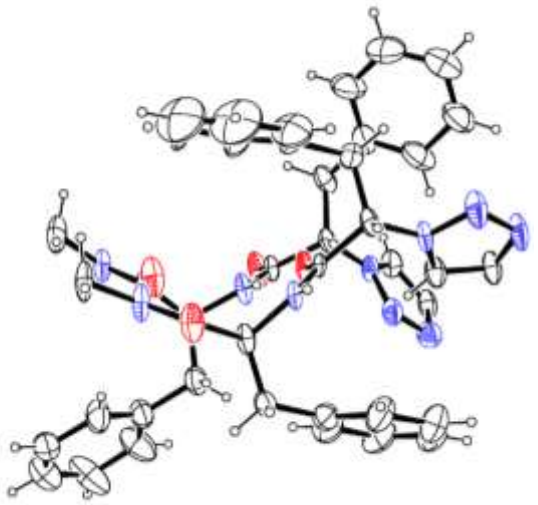

Supplementary Figure 5. ORTEP diagram of PP-II with ellipsoids drawn at 30% probability level.

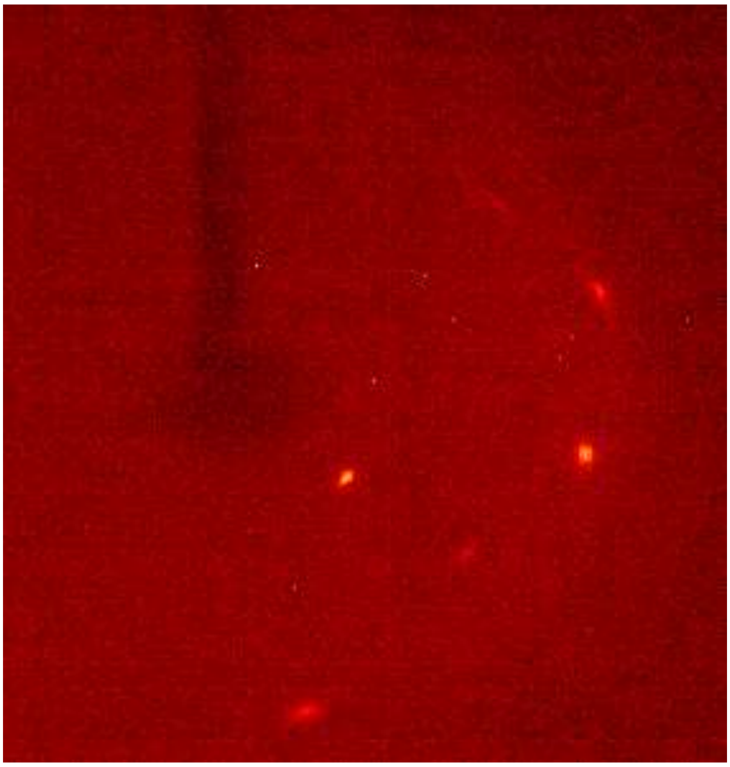

Supplementary Figure 6. Diffraction pattern of PP-III.

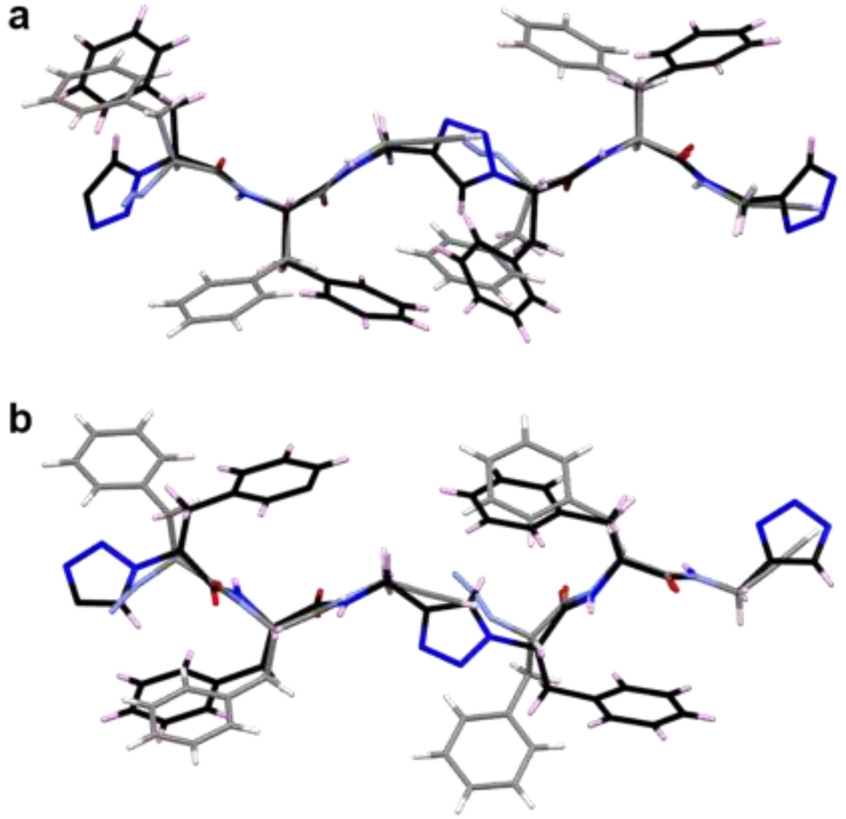

Supplementary Figure 7. **Overlay of crystal structures of DP-II and PP-II**. Overlay of **DP-II** (faded) and the corresponding pseudoprotein chains of **PP-II** (dark), **PP-IIA** (**a**) and **PP-IIB** (**b**)

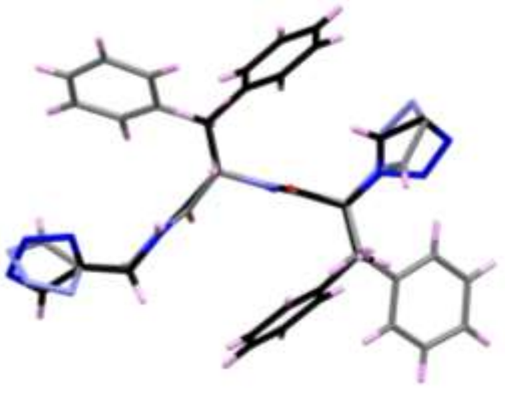

Supplementary Figure 8. **Overlay of the two pseudoprotein chain conformers in PP-II**. Overlay of the two symmetry independent molecules (**PP-IIA** (faded) **PP-IIB** (dark)) in the asymmetric unit of **PP-II**.

Supplementary Table 1. Crystal data of **DP-II, DP-III (**from *o*-xylene gel) and **PP-II**

Parameters	**DP-II**	**DP-III (*o*-xylene)**	**PP-II**
CCDC No	1914914	1914915	1914919
Empirical formula	$C_{21}H_{21}N_5O_2$	$C_{21}H_{21}N_5O_2$	$C_{21}H_{21}N_5O_2$
Formula weight	375.43	375.43	375.43
Temperature	293(2)	296(2) K	293(2) K
Wavelength	0.71073	0.71073 Å	0.71073 Å
Crystal system	monoclinic	Monoclinic	Monoclinic
Space group	$P2_1$	$P\ 2_1$	$P\ 2_1$
Unit cell dimensions	a = 10.64 Å, α = 90°<br>b = 23.461 Å, β = 101.28°<br>c = 12.211 Å, γ = 90°	a = 4.8412(9) Å α = 90°.<br>b = 23.520(4) Å β = 98.041(6)°.<br>c = 8.9243(16) Å γ = 90°.	a = 9.63(6) Å α = 90°.<br>b = 21.38(14) Å β = 102.27(7)°.<br>c = 10.18(7) Åγ = 90°.
Volume	2989.4 $Å^3$	1006.2(3) $Å^3$	2049(23) $Å^3$
Z	6	2	4
Density (calculated)	1.251 g cm^{-3}	1.239 g/cm^{-3}	1.217 g/cm^{-3}
Absorption coefficient	0.084 mm^{-1}	0.083 mm^{-1}	0.082 mm^{-1}
F(000)	1188	396	792
Crystal size	0.15 x 0.15 x 0.05 mm^3	0.21 x 0.15 x 0.10 mm^3	0.25 x 0.15 x 0.10 mm^3
Theta range for data collection	1.700 to 25.000°.	2.883 to 25.998°.	1.905 to 24.995°.
Index ranges	-12<=h<=12,<br>-27<=k<=27,<br>0<=l<=14	-5<=h<=5,<br>-29<=k<=29,<br>-11<=l<=11	-11<=h<=6, -25<=k<=25, -12<=l<=12
Reflections	8151	14296	18962

collected			
Independent reflections	2584 [R(int) = 0.2210]	3922 [R(int) = 0.0436]	7223 [R(int) = 0.1139]
Completeness to theta = 25.242°	99.7 %	99.8 %	99.9 %
Absorption correction	Semi-empirical from equivalents	Semi-empirical from equivalents	Semi-empirical from equivalents
Max. and min. transmission	0.996 and 0.988	0.992 and 0.983	0.992 and 0.980
Refinement method	Full-matrix least-squares on F^2	Full-matrix least-squares on F^2	Full-matrix least-squares on F^2
Data / restraints / parameters	10151 / 1 / 758	3922 / 133 / 254	7223 / 75 / 481
Goodness-of-fit on F^2	1.019	1.019	0.976
Final R indices [I>2sigma(I)]	R1 = 0.0523, wR2 = 0.1223	R1 = 0.0502, wR2 = 0.1084	R1 = 0.0944, wR2 = 0.1934
R indices (all data)	R1 = 0.2208, wR2 = 0.2215	R1 = 0.0967, wR2 = 0.1289	R1 = 0.2773, wR2 = 0.2873
Absolute structure parameter	0.3(10)	1.0(9)	2.4(10)
Extinction coefficient	n/a	0.022(5)	n/a
Largest diff. peak and hole	0.187 and -0.146 e.Å^{-3}	0.242 and -0.135 e.Å^{-3}	0.249 and -0.211 e.Å^{-3}

Supplementary Table 2. Crystal data of **DP-III** from *m*-xylene, chlorobenzene and benzene gels.

Parameters	**DP-III (*m*-xylene)**	**DP-III (chlorobenzene)**	**DP-III (benzene)**
CCDC No	1914917	1914918	1914916
Empirical formula	$C_{21}H_{21}N_5O_2$	$C_{21}H_{21}N_5O_2$	$C_{21}H_{21}N_5O_2$
Formula weight	375.43	375.43	375.43
Temperature	273(2)	296(2) K	293(2) K
Wavelength	0.71073	0.71073 Å	0.71073 Å
Crystal system	monoclinic	Monoclinic	Monoclinic
Space group	$P2_1$	$P\ 2_1$	$P\ 2_1$
Unit cell dimensions	a = 4.8334(14) Å, α = 90°<br>b = 23.536(7) Å, β = 98.021°<br>c = 8.912(3) Å, γ = 90°	a = 4.8381(12) Å α = 90°.<br>b = 23.623(6) Å β = 98.117(6)°.<br>c = 8.901(2) Åγ = 90°.	a = 4.84(6) Å α = 90°.<br>b = 23.6(3) Å β = 97.8(2)°.<br>c = 8.94(12) Åγ = 90°.
Volume	1003.9(5) $Å^3$	1007.1(4) $Å^3$	1010(23) $Å^3$
Z	2	2	2
Density (calculated)	1.242 g cm^{-3}	1.238 g cm^{-3}	1.234 g cm^{-3}
Absorption coefficient	0.083 mm^{-1}	0.083 mm^{-1}	0.083 mm^{-1}
F(000)	396	396	396
Crystal size	0.20 x 0.15 x 0.12 mm^3	0.18 x 0.15 x 0.12 mm^3	0.15 x 0.15 x 0.08 mm^3
Theta range for data collection	2.308 to 25.998°.	2.311 to 25.992°.	2.300 to 24.982°.
Index ranges	-5<=h<=5,<br>-29<=k<=29,<br>-10<=l<=10	-5<=h<=5,<br>-29<=k<=29,<br>-10<=l<=10	-5<=h<=5, -28<=k<=28, -10<=l<=10

Reflections collected	10747	11907	11419
Independent reflections	3918 [R(int) = 0.0472]	3931 [R(int) = 0.0324]	3560 [R(int) = 0.0852]
Completeness to theta = 25.242°	99.8 %	99.9 %	99.9 %
Absorption correction	Semi-empirical from equivalents	Semi-empirical from equivalents	Semi-empirical from equivalents
Max. and min. transmission	0.990 and 0.984	0.990 and 0.985	0.988 and 0.991
Refinement method	Full-matrix least-squares on F^2	Full-matrix least-squares on F^2	Full-matrix least-squares on F^2
Data / restraints / parameters	3918 / 1 / 253	3922 / 1 / 253	3560 / 1 / 262
Goodness-of-fit on F^2	1.018	1.024	0.957
Final R indices [I>2sigma(I)]	R1 = 0.0594, wR2 = 0.1447	R1 = 0.0466, wR2 = 0.1102	R1 = 0.0622, wR2 = 0.1357
R indices (all data)	R1 = 0.1046, wR2 = 0.1669	R1 = 0.0744, wR2 = 0.1238	R1 = 0.1637, wR2 = 0.1812
Extinction coefficient	n/a	n/a	n/a
Largest diff. peak and hole	0.208 and -0.176 e.Å^{-3}	0.221 and -0.152 e.Å^{-3}	0.153 and -0.161 e.Å^{-3}

Supplementary Table 3. Non-covalent interactions in **DP-I, DP-II** and **DP-III**.

Interaction	D-H…A	Symmetry code	**H…A (Å)**/ vdW-d (Å) / *D-H...A (°)*		
			**DP-I**	**DP-II**	**DP-III**
N-H…O	N4-H4…O1	1+x,y,z	**2.11**/0.61/*154.4*		
		1+x,y,z			**2.10**/0.62/*161.4*
	N5-H5…O2	-1+x,y,z	**2.04**/0.68/*164.7*		
		1+x,y,z			**1.99**/0.74/*178.3*
	N4A-H4A…O1C	-1+x,y,-1+z		**2.10**/0.62/*160.5*	
	N4B-H4B…O1A	x,y,z		**2.10**/0.62/*160.9*	
	N4C-H4C…O1B	x,y,z		**2.11**/0.61/*160.1*	
	N5A-H5A…O2B	x,y,z		**1.99**/0.73/*176.2*	
	N5B-H5B…O2C	x,y,z		**2.00**/0.72/*175.1*	
	N5C-H5C…O2A	1+x,y,1+z		**1.98**/0.74/*176.7*	
C-H…O	C1-H1…O1	1+x,y,z	**2.64**/0.08/*132.2*		
		-1+x,y,z			**2.51**/0.21/*141.5*
	C3-H3…O2	1+x,y,z	**2.64**/0.08/*143.1*		
		-1+x,y,z			**2.68**/0.04/*143.3*
	C1A-H1A…O1C	-1+x,y,-1+z		**2.50**/0.23/*141.5*	
	C1B-H1B…O1A	x,y,z		**2.53**/0.19/*140.9*	
	C1C-H1C…O1B	x,y,z		**2.50**/0.22/*140.0*	
	C3A-H3A…O2B	x,y,z		**2.67**/0.05/*143.6*	
	C3B-H3B…O2C	x,y,z		**2.69**/0.03/*143.5*	
	C3C-H3C…O2A	1+x,y,1+z		**2.64**/0.08/*144.3*	
C-H… π	C5B-H5B1… C7A	x,y,z		**2.91**/0.01/*157.4*	

	C5C-H5C1…C7B	x,y,z		**2.91**/0.01/157.4	
	C5A-H5A2…C7C	-1+x,y,-1+z		**2.92**/0.02/158.5	
	C5-H5A…C7	-1+x,y,z			**2.88**/0.02/*158.6*
	C7-H7…N1	2-x,-1/2+y,1-z			**2.67**/0.08/*163.9*
	C7A-H7A…N1A	1-x,-1/2+y,1-z		**2.68**/0.07/*163.3*	
	C7B-H7B…N1C	2-x,-1/2+y,2-z		**2.67**/0.08/*166.5*	
	C7C-H7C…N1B	2-x,-1/2+y,2-z		**2.62**/0.13/*163.6*	

Supplementary Table 4. Non-covalent interactions in **PP-I, PP-II**.

Interaction	D-H…A	Symmetry code	**H…A (Å)**/ vdW-d (Å) / *D-H…A (°)*	
			**PP-I**	**PP-II**
N-H…O	N4-H4…O2	-1+x,y,z	**2.07**/0.65/*170.4*	
	N4A-H4A…O1B	x,y,z		**2.09**/0.63/*167.9*
	N4B-H4B…O1A	1+x,y,z		**2.12**/0.61/*156.7*
	N5-H5…O1	1+x,y,z	**2.14**/0.58/*166.3*	
	N5A-H5A…O2B	1+x,y,z		**2.08**/0.64/*171.0*
	N5B-H5B…O2A	x,y,z		**2.03**/0.69/*160.0*
C-H…O	C1-H1…O1	-1+x,y,z	**2.49**/0.23/150.6	
	C1A-H1A…O1B			**2.32**/0.40/*153.5*
	C1B-H1B…O1A	1+x,y,z		**2.44**/0.28/*150.5*
	C3-H3…O2	1+x,y,z	**2.65**/0.07/*133.0*	
	C3A-H3A…O2B			**2.57**/0.15/*145.4*
C-H… π	C15-H15… π	-1+x,y,z	**2.87**/0.10/*143.7*	
C-H…N	C7-H7…N2	1+x,y,z	**2.45**/0.3/*123.2*	
	C7A-H7A…N2B	1+x,y,z		**2.62**/0.13/*159.7*
	C10A-H10A…N1B	1-x,1/2+y,1-z		**2.64**/0.11/*140.7*
	C7B-H7B…N2A	x,y,z		**2.67**/0.06/*130.1*
O-H…O	O1’…O2’	-1/2-x,2-y,-1/2+z	**2.70**/0.34/---	

Supplementary Table 5. Solubilities of the three polymers **PP-I**, **PP-II** and **PP-III** in different solvents.

Solvent	Solubility (g mL^{-1})		
	**PP-I**	**PP-II**	**PP-III**
N,N-Dimethyl sulfoxide	Insoluble	0.4	0.32
N,N-Dimethyl formamide	Insoluble	Insoluble	0.3
N-Methyl-2-pyrrolidone	Insoluble	Insoluble	0.27
Acetonitrile	Insoluble	Insoluble	Insoluble
Methanol	Insoluble	Insoluble	Insoluble
Water	Insoluble	Insoluble	Insoluble

Supplementary References

1. Sroka-Bartnicka, A., Ciesielski, W., Libiszowski, J., Duda, A., Sochacki, M. & Potrzebowski, M. J. Complementarity of solvent-free MALDI TOF and solid-state NMR spectroscopy in spectral analysis of polylactides. *Anal. Chem*. **82**, 323-328 (2010).
